# A Comprehensive Evaluation of the Activity and Selectivity Profile of Ligands for RGD-binding Integrins

**DOI:** 10.1038/srep39805

**Published:** 2017-01-11

**Authors:** Tobias G. Kapp, Florian Rechenmacher, Stefanie Neubauer, Oleg V. Maltsev, Elisabetta A. Cavalcanti-Adam, Revital Zarka, Ute Reuning, Johannes Notni, Hans-Jürgen Wester, Carlos Mas-Moruno, Joachim Spatz, Benjamin Geiger, Horst Kessler

**Affiliations:** 1 Institute for Advanced Study and Center for Integrated Protein Science, Department of Chemistry, Technische Universität München, Lichtenbergstr. 4, 85747 Garching, Germany; 2 Max-Planck-Institute for Medical Research, Department of Biointerface Science and Technology, Heidelberg, Postal address: Heisenbergstr. 3, 70 569 Stuttgart, Germany; 3 Department of Molecular Cell Biology, Weizmann Institute of Science, Rehovot, 7610001, Israel; 4 Clinical Research Unit, Department of Obstetrics & Gynecology, Technische Universität München, Ismaninger Strasse 22, 81675 Munich, Germany; 5 Pharmaceutical Radiochemistry, Technische Universität München, Munich, Germany; 6 Biomaterials, Biomechanics and Tissue Engineering Group, Department of Materials Science and Metallurgical Engineering, and Centre for Research in NanoEngineering (CRNE), Technical University of Catalonia, 08028-Barcelona, Spain

## Abstract

Integrins, a diverse class of heterodimeric cell surface receptors, are key regulators of cell structure and behaviour, affecting cell morphology, proliferation, survival and differentiation. Consequently, mutations in specific integrins, or their deregulated expression, are associated with a variety of diseases. In the last decades, many integrin-specific ligands have been developed and used for modulation of integrin function in medical as well as biophysical studies. The IC_50_-values reported for these ligands strongly vary and are measured using different cell-based and cell-free systems. A systematic comparison of these values is of high importance for selecting the optimal ligands for given applications. In this study, we evaluate a wide range of ligands for their binding affinity towards the RGD-binding integrins αvβ3, αvβ5, αvβ6, αvβ8, α5β1, αIIbβ3, using homogenous ELISA-like solid phase binding assay.

Structural and signalling responses of cells are tightly regulated by multiple adhesive interactions with the pericellular microenvironment, which promotes the physical networking of neighbouring cells and physical attachment to diverse extracellular matrix (ECM) networks. In addition, multiple environmental cues are mediated via adhesion receptors that bind selectively to external ligands and activate transmembrane signaling pathways that affect cell shape, dynamics, and fate^1^,^2^,^3^.

Integrins are a highly diversified class of key ECM adhesion receptors, that play essential biological functions in all higher organisms. They consist of two distinct transmembrane subunits, one α and one β, which connect the intracellular cytoskeleton and the pericellular ECM. As bidirectional signaling machines integrins respond to environmental cues (outside-in signaling) and at the same time, transduce internal signals (e.g. mechanical stress) to the matrix (inside-out signaling), thereby playing crucial roles in cell-cell communication and ECM^4^. In 1984, Pierschbacher and Ruoslahti discovered the Arg-Gly-Asp (RGD) sequence in fibronectin as the minimal integrin binding motif^5^. Later, this sequence was found in other cell adhesive ECM proteins and described as a common cell recognition motif. These findings were readily followed by the development of multiple peptidic and non-peptidic RGD-based integrin ligands, with various degrees of specificity^6^,^7^,^8^,^9^. To date, eight of the 24 known human integrin heterodimers were shown to bind the RGD-recognition sequence^10^,^11^. Yet, despite their apparent similarity, these integrins can readily distinguish between different RGD-containing ECM proteins (e.g. vitronectin, fibronectin, fibrinogen etc.), and respond differently to the interaction with each one of them.

Given the involvement of integrin-mediated adhesion in the regulation of multiple physiological processes^12^ (e.g. cell migration, proliferation, survival, and apoptosis) as well as pathological processes (e.g. tumor invasion, metastasis), the development of integrin sub-type-exclusive antagonists is highly desirable. Indeed, integrin antagonists were shown to have high therapeutic potential^13^,^14^,^15^,^16^,^17^. Specifically, selective integrin ligands were widely used to target integrin-overexpressing tumors, as inhibitors of cancer angiogenesis^18^,^19^ and as blockers of excessive blood coagulation^15^. Modified integrin ligands were also used for carrying radionuclei or dyes for tumor diagnosis (using PET, SPECT or fluorescent probes)^20^, or for functionalization of adhesive surfaces and development of cell instructive biomaterials^21^,^22^,^23^,^24^.

## Development of integrin subtype-selective compounds

**Figure 1:**
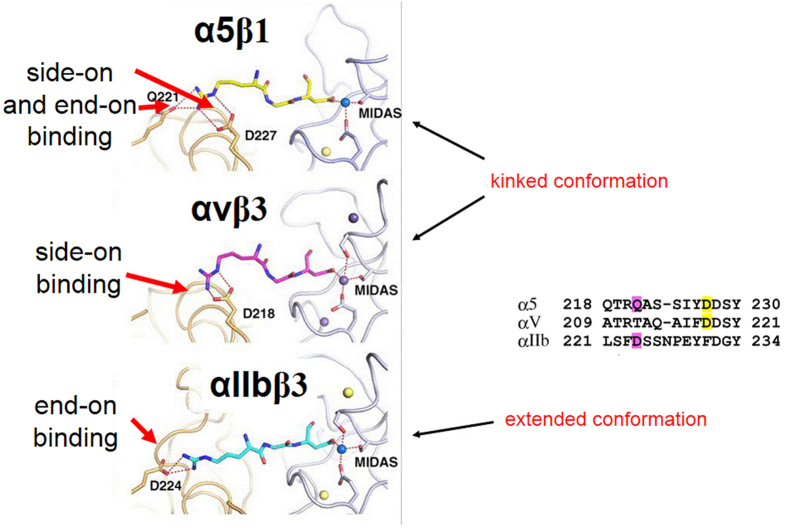
Illustration of different binding modes of a linear RGD peptide to different integrin subtypes. Crystal structures of α5β1 (top), αvβ3 (middle), and αIIbβ3 (bottom) in complex with RGD ligands. Figure adapted from^45^.

Most ECM proteins display a very broad pattern of integrin binding activity. For example fibronectin preferentially binds to α5β1, αvβ6, αvβ8 and to αIIbβ3, although with different activities, while integrin αIIbβ3 is primarily expressed on platelets and binds to specific adhesive proteins, such as fibrinogen/fibrin, prothrombin and plasminogen. Nevertheless, despite their narrow specificity, integrin ligands that target αIIbβ3, should be used for therapeutic purposes with great care, since their excessive systemic administration might cause hemorrhagic disorders. On the other hand, short linear peptides, mimicking the RGD sequence showed a significantly lower binding to αIIbβ3, and had limited effect on platelet functions^5^. A few years later, we addressed the need of focusing on high affinity ligands toward αvβ3 while maintaining selectivity over αIIbβ3, by using cyclic RGD and incorporating one d-amino acid. The latter modification, based on a process called: “spatial screening”^25^,^26^,^27^,^28^, had a drastic impact on the backbone conformation, that changed the selectivity and affinity pattern of the cyclic peptides. These studies revealed that ligands presenting the RGD motif in an extended conformation with distances of 0.7–0.9 nm between the positively-charged arginine residue and the carboxyl group of aspartate, bind preferentially to αIIbβ3^29^. In contrast, if the binding motif is more bent or kinked (as is the situation with the cyclic pentapeptide *c*(RGDf(*N*Me)Val) (=***Cilengitide***)^30^,^31^, ligands tend to bind preferably to other subtypes, such as αvβ3 and α5β1. The crystal structures of integrin antagonists docked into the αvβ3 or αIIbβ3 receptor pocket^32^,^33^,^34^, explained and corroborated this phenomenon, in retrospect (see Fig. 1 and discussion below).

Another crucial aspect of achieving selectivity for αIIbβ3, is the substitution of the guanidine group in the ligand by an amine. In all RGD-binding subtypes except for αIIbβ3, the guanidine group is bound via bifurcated salt bridges to the α-subunit (see Fig. 1), and an amine is not recognized. The binding mode in αIIbβ3 is slightly different and thus, Arg-to-Lys substitution leads to a strong enhancement of the selectivity for αIIbβ3. Nature has developed this alteration, resulting in obtaining selective αIIbβ3-specific ligands, avoiding crosstalk with other integrin subtypes.

Due to the similarity of the RGD binding regions in most integrins, it is not straightforward to achieve high selectivity and, at the same time, high affinity of small synthetic ligands, for distinct subtypes. In fact, most of the ligands described so far as subtype-selective have residual, yet significant affinity to other integrins as well. Recently, we, as well as others, were able to develop ligands with sufficient activity and selectivity to effectively discriminate between two closely related integrin subtypes, such as αvβ3 and α5β1^35^,^36^,^37^ or αvβ6^38^. Their functionalization enabled the selective imaging of αvβ3- or α5β1-expressing tumors in a mouse model, and the differential cell binding on different surfaces^39^,^40^. These molecules were developed by ligand oriented molecular design and later refined on the basis of X-ray structures of the integrins αvβ3 and αIIbβ3^32^,^33^,^41^ and the homology model for α5β1^42^,^43^,^44^. It is interesting to note that the later published crystal structure of α5β1 structurally confirmed the selectivity described for these ligands^45^.

Both, the αvβ3- and the α5β1-selective ligands bind the integrins via the kinked RGD motif, but differences are found in the binding mode of the arginine side chain. As illustrated in Fig. 1, which depicts a linear RGD-ligand in the binding pocket, the guanidine group of arginine is binding in a side-on manner to the Asp218 of the α-subunit of αvβ3, forming a bidendate salt bridge. In addition to this side-on interaction (Asp227 in α5), an end-on interaction of guanidine and Gln221 can be observed in the crystal structure of α5β1^45^. Keeping this difference in mind, the selectivity of the ligands can be explained as follows: the amino pyridine in ***sn243***interacts side-on with the αv-pocket but does not allow an end-on binding due to sterical hindrance, thus reducing strongly the α5β1 affinity. On the other hand, ***44b*** has a full guanidine function allowing side-on and end-on interactions. Recently, we described a way how this small difference between the αv- and the α5-binding pocket can be utilized to design selective peptidic integrin ligands. By alkylation of the *N*_ω_ of the guanidine group of arginine, the α5-specific *end-on* interaction is blocked, leading to a shift in selectivity for αv-integrins^46^.

Apart from targeting integrins αvβ3, α5β1, or αIIbβ3, other clinically relevant integrin subtypes have been explored^16^. For instance, several linear peptides, containing a helical DXLLX motif, were shown to selectively bind αvβ6 and αvβ8, and display low affinity towards all other subtypes. The biological role of αvβ6 and αvβ8 is quite similar, as they are both participating in the activation of transforming growth factor-β (TGF-β) by interacting with the same endogenous ligands TGF-β1 and TGF-β3^47^. Finally, αvβ3 and αvβ5 share very similar biological roles (stimulate angiogenesis), but they perform this task via different mechanisms^48^. Nevertheless, their close structural similarity hampers the development of selective ligands to these integrins^49^,^50^.

### The aims of this work

Since the discovery and first application of integrin-binding RGD peptides in the 1980s, and based on their great impact in medicine, biology, and biophysical sciences, the design and use of synthetic integrin ligands attracts much attention. Most of the current research is focused on the discovery of new integrin-selective ligands and their use for drug delivery, diagnosis, and tumor imaging, which are crucial for developing effective personalized medical platforms.

However, unequivocally ascribing a specific biological role to one integrin receptor remains problematic. Even when high binding affinity towards one distinct integrin subtype is achieved, it often remains unclear whether the observed biological effect is not based on a residual effect on another subtype. This may be attributed to the fact that most studies so far have only focused on the selectivity between a reduced subset of integrins, e.g. αvβ3 vs. αIIbβ3, or αvβ3 vs. α5β1, but have totally neglected the influence of other closely related integrins of the RGD-binding family. This is particularly problematic as integrin expression strongly depends on cell and tissue type, crosstalk within distinct integrin subtypes, time point of study, and biological environment (e.g. tissue type). Last but not least, the activities reported for integrin ligands are usually evaluated using different experimental protocols and are, thus, highly variable.

Consequently, no reliable comprehensive comparison of the IC_50_-values of biologically prominent integrin ligands has been made, so far. Newly designed integrin ligands have seldom been evaluated for their selectivity against a full panel of RGD-binding integrin subtypes, mainly because there were no reliable testing systems established.

In this work, we have evaluated a large number of well-known and widely used integrin-targeting molecules using the same standardized competitive ELISA-based test system, by measuring the inhibition (i.e. IC_50_ values) of integrin binding to immobilized natural ECM ligands. In order to facilitate a direct comparison, we show here (Tables 1 and 2) the affinity values determined with our test system, which always contained reference compounds to standardize the biological data. For some ligands, IC_50_ values were already reported in the literature and the inhibitory activities might slightly deviate from our present data. However, we consider that a direct comparison under identical conditions is very important and thus only represent the data determined during this work. This study includes RGD-based linear and cyclic peptides, peptide-mimetics as well as commonly used reference compounds. Furthermore, in this study we demonstrate, with carefully selected molecules, how functionalization of integrin ligands (e.g. with chelators, anchoring groups) can affect their binding affinity and selectivity. The investigated integrin subtypes studied here include: αvβ3 and αvβ5 (both binding to vitronectin), αvβ6 and αvβ8 (binding to LAP), α5β1 (binding to fibronectin) and αIIbβ3 (binding to fibrinogen), which are all RGD-binding (the only missing RGD-binding integrins are αvβ1 and α8β1, which could not be screened in our test system) and have relevant clinical implications. Since none of the presented ligands have previously been evaluated against such an exhaustive panel of integrin subtypes, the results of this study will provide unprecedented insights into the binding and selectivity profiles of synthetic integrin ligands, thus being of great value for the further development of integrin inhibitors for medical applications. In general, these binding activities correlate very strongly with the inhibition of signal transduction and with the binding affinity of the biochemically highly complex focal adhesions to ECM proteins.

#### An overview of the main approaches for testing integrin subtype-specific ligands

For the development and optimization of biologically active integrin ligands it is of utmost importance to use a reliable and reproducible test system, which yields values for biological activity with low statistical variance and high precision. A careful revision of previously published integrin-binding affinity data for well-known integrin ligands seems to indicate clear differences between the methods used. In general, cell-based methods are strongly dependent on the experimental condition of the study. Thus, the affinity data obtained for the same compound and the same integrin may greatly vary in different cell-based studies. In contrast, non-cellular systems, which are based on the use of isolated integrin receptors, tend to exhibit better biochemical precision and reproducibility. The major drawback of these methods, however, is the fact that they represent a simplified and artificial system, which does not fully mimic the intricate nature of integrin-ligand interactions and the subsequent response of the adhesome-associated signaling. Thus, an efficient combination of both systems is highly recommended for an optimal and efficient development of integrin-targeting drugs. In the following section, several cellular and non-cellular tests are briefly described.

***In vitro*****cellular tests** have been widely used to obtain integrin affinity data. A well-established and commonly used method is based on the concentration-dependent inhibition of cell attachment to a surface that is usually coated with the native cell adhesive proteins^5^. Prior to the test, cells are plated on a surface and afterwards incubated with the soluble compound in different concentrations. Alternatively, cells are incubated in the presence of the integrin ligand, which blocks their attachment to the surfaces. For the evaluation, the attached cells are viewed by transmitted light microscopy, by fluorescence microscopy (in cases when the cells are tagged), or upon use of other functional imaging approaches. Such tests can be performed with a variety of cell types (e.g. NRK-epithelial cells, MG-63, MDA-MB-435^51^ or REF52^52^), as well as with platelets isolated from platelet-rich plasma. Another popular cell-based technique is the calculation of integrin binding affinities based on ligand binding assays^53^. Suspended or adherent cells are incubated with increasing concentrations of an integrin ligand, and afterwards with a radiolabeled ligand that also shows integrin-binding affinity, such as ^125^I-Echistatin or ^125^I-*c*(RGDyK). The radioligand is therefore competing with the compound for binding to the integrin receptors on the cell surface and serves as an internal standard reference for the binding affinity. As described for the cell adhesion assay, a great choice of integrin expressing tumor cell lines as well as epithelial cells are available, such as 293-b β3^54^ or U87MG glioblastoma^55^.

Cell-based tests hold great potential to evaluate not only ligand binding but also its capacity to trigger biological responses relevant for the physiological context. This holds true at least for cases in which the “reporter” cell type is physiologically relevant. It is noteworthy that cellular tests possess some serious intrinsic limitations that are of particular importance during drug development. The major drawback is the limited control over homogeneity in the levels of integrin expression present in the different cell lines used, or even in individual cells in the tested population. Typically, there is one highly overexpressed integrin subtype presented on the surface, but there may also be other minor populated integrin subtypes to which the tested compounds may bind. It is even more problematic that these subtype expression levels may change over time. In addition, the total surface receptor density can strongly vary depending on specific conditions (number of passages in culture, presence of other integrins, cell culture conditions, state of cell cycle etc.). For example, a phenomenon known as “integrin crosstalk” has been proven for αvβ3 and α5β1. Specifically, α5β1 integrin was shown to modulate (up or down) the expression of another subtype and thus significantly alters the expression pattern^56^. Consequently, the comparison of affinity data measured for different cell lines is highly challenging.

**Figure 2:**
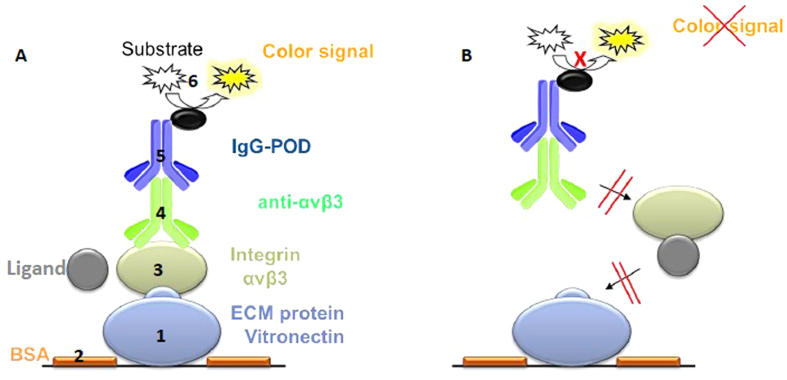
Schematic illustration of the enzyme-linked immunosorbent assay (ELISA). (**A**) **1**. Each well (96-well plate) is coated with an ECM protein (e.g. vitronectin for αvβ3). **2**. Uncoated surface is blocked by bovine serum albumin (BSA). **3.** ECM protein competes with the tested ligand for binding to the soluble integrin (e.g. αvβ3). **4.** Integrin bound to ECM protein is detected by an integrin-specific primary antibody. **5.** Secondary antibody, conjugated with a peroxidase (POD), detects bound primary antibody. **6.** Peroxidase converts a colorless substrate into a colored substrate (TMB, 3,3′,5,5′-tetrametylbenzidine). (**B**) The ligand inhibits binding of the coated ECM protein to the integrin. Consequently, steps 3–6 are blocked and no color signal can be detected.

**Tests carried out in cell-free systems,** use isolated extracellular domains of integrins in conjunction with ECM proteins, either extracted from human tissue or produced by recombinant methods. Most of these tests are based on competitive solid phase binding assays, in which one component is bound to the multi-well plate and in a subsequent step, soluble ligands are added, testing their capacity to block the binding to that component. Mainly two procedures are described in the literature, differing in the molecule used to coat the surfaces: the isolated integrin extracellular domains or the ECM protein. In the first case, the integrin is coated on the surface, followed by an incubation with a mixture of the native ECM protein and increasing concentrations of the ligand of interest^49^. In an alternative test system, the natural ECM protein (vitronectin for αvβ3 and αvβ5, LAP (TGFβ) for αvβ6 and αvβ8, fibronectin for α5β1 and fibrinogen for αIIbβ3) is immobilized onto the surface, and the soluble integrin, together with a serial dilution of the inhibitory ligand, is added afterwards^57^. The read-out in both procedures is usually done in an ELISA-like manner by using conjugated antibodies recognizing the integrin head groups. A detailed schematic illustration of the different steps of the integrin binding assay is presented in Fig. 2. In comparison to many other test systems, this system allows the accurate (SD~10%) and reproducible determination of IC_50_ values for almost all RGD-binding integrin subtypes. For a detailed description of the reference to the SI. As the quality of the integrins strongly depend on the batch and providers a reference compound always have to be used for each test plate as internal standard (we used: ***Cilengitide***, *c*(RGDf(*N*Me)V) (αvβ3–0.54 nM, αvβ5–8 nM, α5β1–15.4 nM), linear peptide ***RTDLDSLRT*** (αvβ6–33 nM; αvβ8–100 nM) and ***tirofiban*** (αIIbβ3–1.2 nM).

Preliminary studies in our laboratories comparing the two methods (surface-bound integrin vs. soluble integrin) revealed significant differences in the antagonistic activity of control ligands in regards to the integrin subtype used. Whereas very similar IC_50_ values were found regardless of the method used for integrins αvβ3 or αvβ5, the activity towards α5β1 seemed to be highly dependent on the experimental protocol. The drug ***Cilengitide*** exhibited very high affinity (in the low nanomolar range) towards soluble α5β1 when fibronectin (i.e. the natural ECM ligand) was immobilized on surfaces. However, coating of the integrin and subsequent incubation of ***Cilengitide*** with fibronectin generally resulted in low antagonistic activities and poor reproducibility within assays (unpublished data). This was already observed in our stem peptide ***c**(**RGDfV***)^26^, and may be attributed to the possible denaturation of α5β1 integrin.

## Results and Discussion

### The gold standard integrin inhibitor, Echistatin

The disintegrin ***Echistatin*** was first isolated in 1988 from snake venom as an effective inhibitor of platelet-fibrinogen interaction, as well as of platelet aggregation^58^. This small folded protein contains an RGD-sequence in a well-exposed loop which was described to bind to αIIbβ3, αvβ3, αvβ5, and α5β1 with very high affinity^59^. Since the Tyr-31 residue of ***Echistatin*** can be labeled with ^125^I using a standard procedure has turned this compound into a commonly used positive control for many competitive cellular integrin binding assays^60^,^61^. We therefore included ***Echistatin*** in our study to determine its selectivity profile and to compare its binding activities to those previously obtained from cellular assays. ***Echistatin*** showed a very broad affinity pattern. It binds to the whole panel of investigated integrins with IC_50_-values in the low nano-molar range (Table 1). As already reported, it shows particularly low IC_50_-values for αvβ3 (0.46 nM), α5β1 (0.57 nM), and αIIbβ3 (0.9 nM). Interestingly, ***Echistatin*** exhibited the lowest IC_50_-values for these integrins compared to all other compounds investigated within this study. Thus, it also represents an ideal candidate to be included as a positive control in cell-free integrin binding tests.

### Linear RGD integrin inhibitory peptides

The RGD-sequence was originally discovered as the minimal binding epitope of fibronectin and has extensively been investigated over the last decades. Moreover, it has been shown that the presence and chemical nature of flanking residues have a strong influence on its activity^5^. Thus, early studies with the RGD-motif were conducted with linear tri- to heptapeptides, based on the sequence found in fibronectin. In our study, the following linear peptides were included: ***RGD**, **RGDS**, **GRGD**, **GRGDS**, **GRGDSP,***and ***GRGDSPK***. In original reports, these peptides were described to bind to αvβ3 and αvβ5, but also showed relatively good IC_50_-values for the integrin subtypes α5β1 and αvβ6, as well as low affinity for αIIbβ3^62^. Apart from the compounds mentioned above, ***GRGDNP***^63^ and ***GRGDTP***^64^ peptides were also included in our test system, as they are frequently used in biological studies. Especially, ***GRGDNP*** has been described to prefer binding to α5β1. In our evaluation, all linear peptides showed the lowest IC_50_-values for the integrin subtype αvβ3 (12–89 nM), with IC_50_-values for αvβ5 ranging from 167 to 580 nM and for α5β1 from 34 to 335 nM. These peptides generally displayed high IC_50_-values towards αvβ6 and αvβ8. More surprisingly, none of the linear peptides exhibited an IC_50_-value below 10 μM on αIIbβ3. These results demonstrate that the linear RGD peptides are active on integrins αvβ3, αvβ5, and α5β1, and selective against αvβ6, αvβ8 and αIIbβ3. As presented in Table 1, the residues flanking the RGD-motif essentially contribute to the binding affinity for αvβ3 (and to a lower extent for α5β1 and αvβ5). In particular, the IC_50_-value to αvβ3 increases 7-fold from the linear tripeptide fragment ***RGD*** (89 nM) to the heptapeptide ***GRGDSPK*** (12.2 nM).

### Cyclic RGD peptides

A major disadvantage of linear peptides is their low stability regarding enzymatic degradation, limiting their applicability for *in vivo* studies^65^. This can be significantly improved by cyclization and incorporation of a d-amino acid residue, as illustrated by cyclic pentapeptides of the formula *c*(RGDxX) and cyclic hexapeptides^66^. Moreover, reduction of the conformational space by cyclization can improve the biological potency of linear peptides when the bioactive conformation is matched^67^. One of the first cyclic compounds that was developed and used in cellular studies was **c**(***RGDfV***) which showed outstanding affinity for αvβ3, while retaining total selectivity against αIIbβ3. This core structure was later modified to develop a series of new ligands with improved activity and selectivity profiles^68^,^69^. Extensive Structure-Activity Relationship (SAR) studies on the model sequence *c*(RGDxX) showed, that the presence of an aromatic amino acid in the d-configuration (i.e. d-Phe, d-Tyr, d-Trp) at the position 4 (residue x) was essential for the αvβ3-binding affinity, whereas the amino acid at position 5 (residue X) had little effect on the biological activity^68^,^69^. Based on the stem peptide *c*(RGDfV), ***Cilengitide**, c*(RGDf(*N*Me)V), the most active cyclic pentapeptide described to date, was developed via a systematic *N*-methylation scan. ***Cilengitide*** has a half-life in man of about four hours and is not metabolized systemically^70^. It became a drug candidate in phase II and III clinical studies for the treatment of different tumors^71^,^72^,^73^,^74^, however failed in phase III as drug against glioblastoma. Despite its extraordinarily low IC_50_-value for αvβ3 and αvβ5, the higher, but still significant value for α5β1 subtype is often neglected^75^. In this regard, our study clearly shows that this compound has also a remarkably low IC_50_-value for α5β1 (14.9 nM). Noteworthy, the IC_50_ for αvβ3 (0.61 nM) and αvβ5 (8.4 nM) were the highest obtained among all synthetic peptides developed and studied. As previously indicated, the valine residue can be substituted by almost any other amino acid^66^. Hence, for biophysical or medical applications where a functionalization or ligation of integrin ligands is needed, the derivative ***c**(**RGDfK***)^76^ is often used. Lys has been found to be a good anchoring point for the attachment of functional units not only because it does not affect the binding affinity of the stem peptide significantly, but also because it easily allows the linkage of other chemical groups via the free amine. A pilot study by us used acrylate-functionalized derivatives of **c**(***RGDfK***) to mediate the adhesion of osteoblasts on a polymethylmetacrylate (PMMA) surface^77^. Since that time, ***c**(**RGDfK***) and also ***c**(**RGDfE***) were functionalized for a large number of biological applications^78^,^79^. Other cyclic penta-peptides used for functionalization are ***c**(**RGDyK***)^80^ as well as ***c**(**RGDfC***)^81^, are both included in our study. Just like the linear peptides, all of the cyclic pentapeptides of the type *c*(RGDxX) tested showed moderate to low IC_50_-values for αvβ3, αvβ5, and α5β1, and no binding to αIIbβ3, which is of major importance for *in vivo* applications. Noteworthy, all the cyclic peptides displayed lower αvβ3 IC_50_-values (i.e. in the range of 1.5 to 6 nM) compared to the linear derivatives, and followed the order ***c**(**RGDfV***) < ***c**(**RGDfK***) < ***c**(**RGDyK***) < ***c**(**RGDfC***). Therefore, cyclic RGD-peptides should be the preferred choice when high αvβ3-binding activities are required. The IC_50_ values for αvβ5 and α5β1 varied from 250 to 503 nM and from 141 to 236 nM, respectively. Interestingly, all the cyclic compounds showed relatively low IC_50_-values for αvβ6 (49–75 nM), which has not been discussed in any of the references so far.

Ruoslahti *et al*. discovered the RGD-containing double cysteine-bridged (1–4, 2–3) peptide ***RGD-4C***^82^ (ACDCRGDCFCG) by phage display and reported a low IC_50_-value towards the subtypes αvβ3 and αvβ5, and specificity over α5β1. This peptide represents one of the most commonly used molecules in cellular tests and in *in vivo* studies and has been conjugated to target αvβ3-overexpressing cells^83^. To reduce the synthetic complexity that arises from two disulfide bridges, Hölig *et al*. developed the single cysteine-bridged peptide ***RGD10*** (GARYCRGDCFDGR)^84^, which has the same IC_50_ value and selectivity properties as the original ***RGD-4C***, and has been functionalized for different applications as well, (e.g. for surfaces coating or targeting liposomes). In our studies, the ***RGD-4C*** peptide exhibited an IC_50_-value for αvβ3 of 8.3 nM, which is comparable to that observed for the best linear RGD-sequences but considerably lower than that of cyclic RGD-containing penta-peptides. Furthermore, it also shows a good value for αvβ5 (46 nM), a high IC_50_-value for αvβ6 and α5β1, and no affinity for αvβ8 and the platelet integrin αIIbβ3. As reported in other studies, ***RGD-10*** exhibits a similar pattern of bioactivity, though with a trend towards increased αvβ3/αvβ5 selectivity: the IC_50_ for αvβ3 remains almost the same, whereas the αvβ5 affinity drops to 102 nM. Based on the ***RGD-4C*** peptide, Indrevoll *et al*. developed a PEGylated bicyclic, mono cysteine-bridged peptide with the sequence KCRGDCFC (***NC100717***)^85^ targeting the αvβ3 and αvβ5 integrin subtype. This scaffold is the targeting unit of functionalized compounds (chelators and dyes), e.g. the ^18^F-labeled compound ^18^F-***AH111858*** (Fluciclatide^86^, GE), described in detail in the section of functionalized molecules. ***NC100717*** showed a low nanomolar IC_50_-value for αvβ3 (1.1 nM) and αvβ5 (41 nM) in our test system. Nonetheless, for application of disulfide-bridged cyclic peptides it may be of interest to consider the stability of disulfide bridges *in vivo*^87^. Finally, another integrin binding motif in fibronectin is the inverse sequence *iso*DGR which is based on the NGR motif after *in situ* rearranging from asparagine to *iso* apartate^88^,^89^. The integrin subtype selectivity strongly varies depending on the flanking residues of the sequence. Recently, the compound ***c***(**phg*****iso*****DGRk**)^50^, which is bi-selective for αvβ6 and α5β1, was identified and used for cellular studies. In our test system, it could be shown here that the compound moreover binds to αvβ8 as well.

### Peptidomimetics and other ligands

The non-RGD linear pentapeptide Ac-PHSCN-NH_2_ was derived from the synergy domain of fibronectin and is clinically developed under the trade name ***ATN161***^90^ for the treatment of several solid tumors as it is highly active for the α5β1 subtype, with some affinity for αvβ3 and αvβ5^16^. Interestingly, ***ATN161*** showed a clear selectivity for α5β1 (4.2 nM) in our testing system, being essentially inactive for all other integrins investigated.

The non-peptidic compound ***JSM6427***^36^ was designed by Stragies *et al*. and later developed in clinical phase for the treatment of age-related macular degeneration as it strongly inhibits neovascularization in the eye. It is described to be a relatively selective α5β1 antagonist, although values for αvβ6 and αvβ8 were not published. Indeed, we found ***JSM6427*** to be tri-selective for α5β1 (2.5 nM), αvβ6 (23 nM), and αvβ8 (8.2 nM).

Recently, we reported the synthesis and binding affinity of the highly active αvβ3-selective ***sn243***^37^ and the α5β1-selective ***44b***^91^ peptidomimetic ligands. In a proof-of-concept study, these compounds were functionalized and used for molecular imaging^92^ as well as for biophysical studies^39^,^40^, showing their potential to discriminate the two integrin subtypes αvβ3 and α5β1 both, *in vitro* and *in vivo*. Here, we also included them to evaluate their full pattern of integrin selectivity, and found, besides the expected high activities for the corresponding subtype (***sn243***: 0.65 nM αvβ3; ***44b***: 2.3 nM α5β1), a low nanomolar IC_50_-value for αvβ8 for ***44b*** (37 nM).

### Integrin αvβ6-binding ligands

The α-helical binding motif DLXXL, an αvβ6 subtype-specific binding motif, was initially discovered by phage display and later shown to be also present in the natural αvβ6 ligand latency associated peptide (LAP). Two compounds containing this motif are included in this study as they have extensively been used for addressing selectively αvβ6-expressing cells *in vivo*, e.g. as targeting unit for molecular imaging. The RTD containing 9-mer peptide ***RTDLDSLRT***^93^ as well as the 20-mer peptide ***A20FMDV2***^94^, which is derived from a foot and mouth disease virus peptide (sequence: NAVPNLRGDLQVLAQKVART), were proven to be subtype-selective in our study and exhibited an IC_50_-value of 29.5 and 0.93 nM for αvβ6, respectively. The peptidomimetic compound ***Mol11***^49^, which was described as the first αvβ6 selective small molecule ligand, was resynthesized for this study as an enantiomerically pure (*S*-enantiomer) compound and indeed showed a very low IC_50_-value for αvβ6 (1.3 nM), but also good values in the lower nanomolar range for αvβ3 (13.2 nM), and αvβ8 (18.5 nM). Recently, we were able to develop the cyclic peptide ***c**(**FRGDLAFp**(**NMe**)**K***)^38^, which mimics the binding epitope of the helical DLXXL-motif. The IC_50_ of this peptide was determined to be 0.28 nM for αvβ6, the highest affinity among the compounds investigated.

### Integrin αIIbβ3-binding ligands

The αIIbβ3 integrin receptor, also known as glycoprotein receptor (GP)-IIb/IIIa, is expressed uniquely on the surface of platelets and megakaryocytes, a type of platelet-producing cells in the bone marrow. By binding to its natural ligand fibrinogen, αIIbβ3 is involved in primary hemostasis during platelet formation. Thus, application of αIIbβ3 ligands was earlier explored as a clinical approach for anti-thrombotic therapy^15^. Today, there are two FDA-approved drugs targeting selectively the αIIbβ3 receptor, Intrifiban (***Eptifibatide***)^95^ and ***Tirofiban***(Aggrastat)^96^, both clinically used for patients with acute coronary syndromes undergoing percutaneous coronary intervention. ***Eptifibatide*** is a cysteine-bridged cyclic RGD-containing hexapeptide. It was first described in 1993 as a potent subtype-selective integrin antagonist and is, as well as the small molecule αIIbβ3 inhibitor ***Tirofiban***, used both clinically and preclinically. Another αIIbβ3 selective compound included in this comparison of integrin ligands is ***GR144053***^97^. It is described as an orally available, highly potent subtype-selective fibrinogen inhibitor and is used in many preclinical studies. In our test system, these three compounds have been confirmed to be selective αIIbβ3 inhibitors. The lowest αIIbβ3 IC_50_-value was determined for ***Tirofiban*** (1.3 nM), followed by ***Eptifibatide*** (2.8 nM) and ***GR144053*** (18 nM).

#### Functionalized compounds

Many of the integrin ligands that are used for biophysical or medical experiments require a functional unit (e.g. for a strong covalent binding to the surface) or a chelator for molecular imaging. For that reason, the bioactive moiety is linked to this functionality via a spacer that separates the two entities of the molecule. Ideally, by using the right anchoring point in the molecule, the loss of activity upon functionalization is low. Nevertheless, the size, lipophilicity, and other parameters like the rigidity of the functional as well as the spacing unit can influence the binding of the bioactive moiety to its target^98^,^99^. To estimate the influence on the IC_50_-values after modification of the ligand, five compounds were chosen as examples (Table 2). Among three of them, ***c**(**RGDfK***), which is functionalized via its lysine side chain, represents the bioactive targeting unit. The IC_50_-value of the unmodified ligand was determined to be 2.3 nM for the αvβ3 integrin subtype (Table 1). After modification to ***F-Galacto-c(RGDfK)***^100^ (for molecular imaging) and ***c(RGDfK)-Peg-MPA***) (MPA = mercapto propionic acid), the IC_50_ moderately decreased to 8 and 15 nM, respectively. Exactly the opposite, namely a better αvβ3 IC_50_ was observed for the (***Ga**)**NOPO-c**(**RGDfK***) (1.1 nM)^101^. This phenomenon could already be observed for previously published compounds and can be explained, inter alia, by charge effects and/or altered van-der-Waals interactions. For example, large substituents like chelators possess a high surface area and can randomly interact via unspecific van-der-Waals interactions with parts of the protein. This weak interaction decreases the k_off_-rate and thus can lead to IC_50_-values compared to the original targeting peptide alone. Concerning the selectivity profile of the modified compounds, no changes are observable. This is also found for the αvβ6/α5β1 bi-selective peptide ***c**(**phgisoDGRk***), where the selectivity is not affected but the IC_50_-value for the two integrins doubles (2-fold) after functionalization to **c**(***phgisoDGRk***)**-Peg-MPA**)^52^. ***Fluciclatide,*** an imaging agent developed by GE Healthcare, consists of ***NC100717*** as targeting unit^84^. For this example, the IC_50_-value for αvβ3 decreased 3-fold and no change in selectivity was observable. To sum up, the functionalization of a bioactive molecule can alter its integrin binding affinity. This means that in principle, every functionalized compound for both *in vitro* and *in vivo* applications should be tested individually to obtain comparable results. It is important to mention here that e.g. the introduction of a PEG spacer does not guarantee better ligands properties (solubility, affinity). Peg is not always extended in aqueous solution and thus the distance between the biomolecule and the functional unit is not defined^99^. Changes in affinity induced by a spacer and a functional unit are hard to predict. Additionally, functionalization of bioactive compounds can also strongly alter the pharmacodynamics, e.g. the total uptake and distribution of the compound in the organs after its intake. Mostly, this is observable because of a big change in lipophilicity due to the different modifications. Especially for molecular imaging, where only defined structures in the body should be visualized, it is important to evaluate and optimize the effect of every modification (e.g. different spacer, chelator, coordinating metal) in this regard. Important points to be addressed regarding the applicability of the probes in standard procedures of diagnosis are the simplicity of production and the flexibility in the type of tracer that can be introduced. In the case of functionalization for surface coating, the strength and stability of the binding to the surface by the anchoring unit and the length and chemical structure of the spacer for a defined purpose (e.g. cell adhesion) has to be taken into account.

#### Molecules used as negative control in the determination of binding activities

For cellular and in vitro experiments, molecules with comparable steric properties and lipophilicity but without biological activity often serve as control compounds. Targeted substitution of any of the three amino acids in the RGD-sequence leads to inactivation of the ligand. A substitution of glycine by alanine leads to steric repulsions on the binding groove between the α- and β-subunit. Moreover, any elongation of the ligand (e.g. glycine to β-alanine and aspartate to glutamate substitution) leads to complete loss of binding activity. The molecules can be functionalized in the same way as their active biologically active counterparts (e.g. via lysine side chain). For this study, linear and cyclic control molecules have been synthesized and evaluated.

## Conclusion

After the initial discovery of the RGD sequence in fibronectin, a large number of integrin ligands, binding to RGD-recognizing integrins, were developed by many groups around the world and became highly important for medical applications and for biophysical studies. For the development of each of these compounds, different evaluation techniques (e.g. various cel-based and cell free methods) have been used, allowing a good comparison and selection process of the compounds within a study. However, comparing the values determined by different groups for the very same compound shows very high deviations. For this reason we evaluated for the first time the most frequently used compounds in a homogenous solid phase binding assay for their binding affinity to six RGD-binding integrins (αvβ3, αvβ5, αvβ6, αvβ8, α5β1, αIIbβ3). This gives the possibility to choose the ligand with the ideal affinity and selectivity pattern for a given application and opens new doors for the application of those ligands. However, the complex mechanism including several steps of conformational transitions in which the initial ligand binding to the resting state of the integrin and the stronger binding in the focal adhesion, might have consequences for the IC_50_-value given here with data under different environmental condition *in vivo*^102^.

## Methods Section

### Integrin Binding Assay

The activity and selectivity of integrin ligands were determined by a solid-phase binding assay according to the previously reported protocol^103^ using coated extracellular matrix proteins and soluble integrins. The following compounds were used as internal standards: ***Cilengitide**, c*(RGDf(*N*Me)V) (αvβ3–0.54 nM, αvβ5–8 nM, α5β1–15.4 nM), linear peptide ***RTDLDSLRT***^4^ (αvβ6–33 nM; αvβ8–100 nM) and ***tirofiban***^5^ (αIIbβ3–1.2 nM).

Flat-bottom 96-well ELISA plates (BRAND, Wertheim, Germany) were coated overnight at 4 °C with the **ECM-protein** (**1**) (100 μL per well) in carbonate buffer (15 mM Na_2_CO_3_, 35 mM NaHCO_3_, pH 9.6). Each well was then washed with PBS-T-buffer (phosphate-buffered saline/Tween20, 137 mM NaCl, 2.7 mM KCl, 10 mM Na_2_HPO_4_, 2 mM KH_2_PO_4_, 0.01% Tween20, pH 7.4; 3 × 200 μL) and blocked for 1 h at room temperature with TS-B-buffer (Tris-saline/BSA buffer; 150 μL/well; 20 mM Tris-HCl, 150 mM NaCl, 1 mM CaCl_2_, 1 mM MgCl_2_, 1 mM MnCl_2_, pH 7.5, 1% BSA). In the meantime, a dilution series of the compound and internal standard is prepared in an extra plate, starting from 20 μM to 6.4 nM in 1:5 dilution steps. After washing the assay plate three times with PBS-T (200 μL), 50 ul of the dilution series were transfered to each well from B–G. Well A was filled with 100 ul TSB-solution (blank) and well H was filled with 50 ul TS-B-buffer. 50 ul of a solution of **human integrin** (**2**) in TS-B-buffer was transfered to wells H–B and incubated for 1 h at rt. The plate was washed three times with PBS-T buffer, and then **primary antibody** (**3**) (100 μL per well) was added to the plate. After incubation for 1 h at rt, the plate was washed three times with PBS-T. Then, **secondary peroxidase-labeled antibody** (**4**) (100 μL/well) was added to the plate and incubated for 1 h at rt. After washing the plate three times with PBS-T, the plate was developed by quick addition of SeramunBlau (50 μL per well, Seramun Diagnostic GmbH, Heidesee, Germany) and incubated for 5 min at rt in the dark. The reaction was stopped with 3 M H_2_SO_4_ (50 μL/well), and the absorbance was measured at 450 nm with a plate reader (POLARstar Galaxy, BMG Labtechnologies). The IC50 of each compound was tested in duplicate, and the resulting inhibition curves were analyzed using OriginPro 7.5G software. The inflection point describes the IC50 value. All determined IC50 were referenced to the activity of the internal standard.

αvβ3

1. 1.0 μg/mL human vitronectin; Millipore.
2. 2.0 μg/mL, human αvβ3-integrin, R&D.
3. 2.0 μg/mL, mouse anti-human CD51/61, BD Biosciences.
4. 1.0 μg/mL, anti-mouse IgG-POD, Sigma-Aldrich.

α5β1

1. 0.5 μg/mL; human fibronectin, Sigma-Aldrich.
2. 2.0 μg/mL, human α5β1-integrin, R&D.
3. 1.0 μg/mL, mouse anti-human CD49e, BD Biosciences.
4. 2.0 μg/mL, anti-mouse IgG-POD, Sigma-Aldrich.

αvβ5

1. 5.0 μg/mL; human vitronectin, Millipore.
2. 3.0 μg/mL, human αvβ5-integrin, Millipore.
3. 1:500 dilution, anti-αv mouse anti-human MAB1978, Millipore.
4. 1.0 μg/mL, anti-mouse IgG-POD, Sigma-Aldrich.

αvβ6

1. 0.4 μg/mL; LAP (TGF-β), R&D.
2. 0.5 μg/mL, human αvβ6-Integrin, R&D.
3. 1:500 dilution, anti-αv mouse anti-human MAB1978, Millipore.
4. 2.0 μg/mL, anti-mouse IgG-POD, Sigma-Aldrich.

αvβ8

1. 0.4 μg/mL; LAP (TGF-b), R&D.
2. 0.5 μg/mL, human αvβ8-Integrin, R&D.
3. 1:500 dilution, anti-αv mouse antihuman MAB1978, Millipore.
4. 2.0 μg/mL, anti-mouse IgG-POD, Sigma-Aldrich.

αIIbβ3

1. 10.0 μg/mL; human fibrinogen, Sigma-Aldrich.
2. 5.0 μg/mL, human platelet integrin αIIbβ3, VWR.
3. 2.0 μg/mL, mouse anti-human CD41b, BD Biosciences.
4. 1.0 μg/mL, anti-mouse IgG-POD, Sigma-Aldrich.

## Additional Information

**How to cite this article**: Kapp, T. G. *et al*. A Comprehensive Evaluation of the Activity and Selectivity Profile of Ligands for RGD-binding Integrins. *Sci. Rep.* **7**, 39805; doi: 10.1038/srep39805 (2017).

**Publisher's note:** Springer Nature remains neutral with regard to jurisdictional claims in published maps and institutional affiliations.

## Supplementary Material

Supplementary Information

## Figures and Tables

**Table 1:**
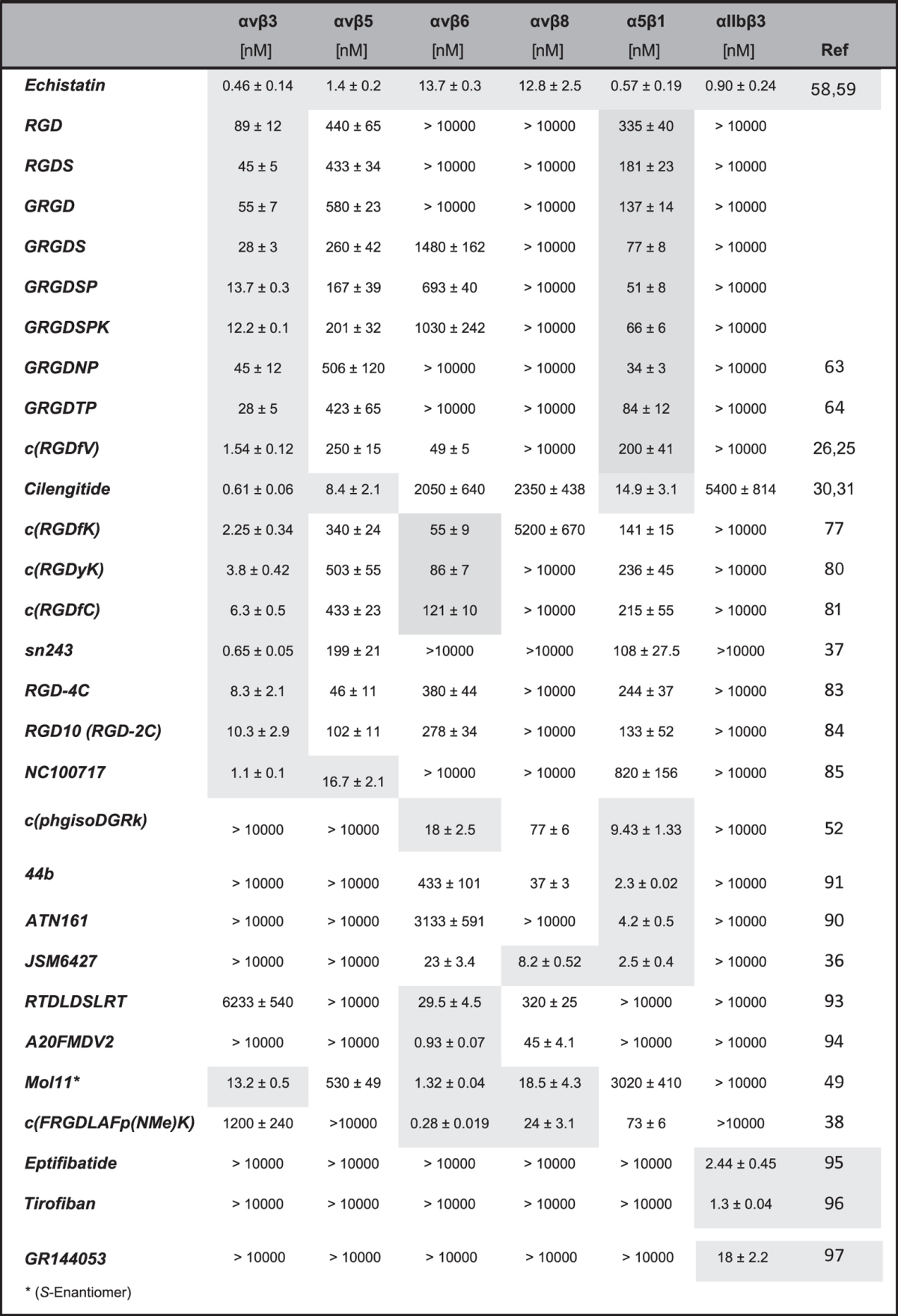
IC_50_-values for the integrin ligands investigated for the subtypes αvβ3, αvβ5, αvβ6, αvβ8, α5β1, and αIIbβ3.

Specificity or subtype with the lowest IC_50_-value are highlighted. All values were referenced as given in the description of the assay and in the SI.^*^(*S*-Enantiomer).

**Table 2:**
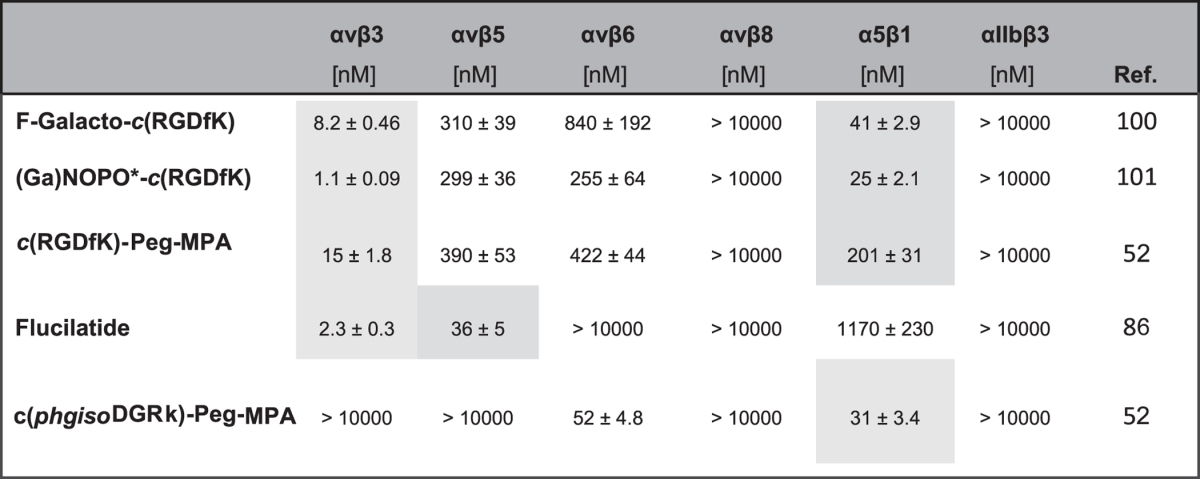
IC_50_-values for the functionalized integrin ligands investigated for the subtypes αvβ3, αvβ5, αvβ6, αvβ8, α5β1 and αIIbβ3.

Specificity or subtypes with the best IC_50_-values are highlighted, respectively. All values were referenced as given in the description of the assay and in the SI.^*^NOPO 1,4,7-triazacyclononane-1,4-bis[methylene(hydroxymethyl)phosphinic acid]-7-[methylene(2-carboxyethyl)phosphinic acid.^**^21-amino-4,7,10,13,16,19-hexaoxaheneicosanoic acid.
