# Supplementary material for: A Comprehensive Evaluation of the Activity and Selectivity Profile of Ligands for RGD-binding Integrins
Source: Sci Rep. 2017 Jan 11;7:39805. doi: 10.1038/srep39805 (PMC5225454; doi:10.1038/srep39805)
Supplement: Supplementary Information [file srep39805-s1.pdf]

Supporting Information

A Comprehensive Evaluation of the Activity and Selectivity Profile of Ligands for RGD-binding Integrins

Tobias G. Kapp, Florian Rechenmacher, Stefanie Neubauer, Oleg V. Maltsev, Ada E. Cavalcanti-Adam, Revital Zarka, Ute Reuning, Johannes Notni, Hans-Jürgen Wester, Carlos Mas-Moruno, Joachim Spatz, Benjamin Geiger & Horst Kessler

**E-mail: kessler@tum.de*

1. General information

Chemicals: All reagents and solvents were obtained from commercial suppliers and used without further purification.

Chromatography: Analytical HPLC-ESI-MS was performed on a Hewlett-Packard Series HP 1100 equipped with a Finnigan LCQ mass spectrometer using a YMC-Hydrosphere C18 column (12 nm pore size, 3 µm particle size, 125 mm×2.1 mm) or YMC-Octyl C8 column (20 nm pore size, 5 µm particle size, 250 mm×2.1 mm) and H_2O (0.1% *v*/*v* formic acid) / MeCN (0.1% *v*/*v* formic acid) as eluents. Semi-preparative HPLC was performed using a Beckmann instrument (system gold, solvent delivery module 126, UV detector 166), an YMC ODS-A column (20×250 mm, 5 µm), flow rate: 8 mL/min, linear gradients of H_2O (0.1% *v*/*v* TFA) and MeCN (0.1% *v*/*v* TFA).

2. Synthesis

General synthetic procedures

GP1 Loading of CTC-resin

Peptide synthesis was carried out using CTC-resin (1.6 mmol/g) following standard Fmoc-strategy. Fmoc-Xaa-OH (1.2 eq.) were attached to the CTC-resin with *N,N*-diisopropylethylamine (DIPEA; 2.5 eq.) in anhydrous DCM (8 mL/g resin) at room temperature for 1 h. The remaining reactive trityl chloride groups were capped by addition of 1mL/g(resin) of a solution of MeOH, DIEA (5:1; v:v) to the solution and after incubation for 15 min. The resin was filtered and washed 5 times with DCM and 3 times with MeOH. The loading capacity was determined by weight after drying the resin under vacuum and ranged from 0.8-1.4 mmol/g.

GP2 On-Resin Fmoc Deprotection

The resin-bound Fmoc peptide was treated with 20% piperidine in NMP (v/v) for 10 minutes and a second time for 5 min. The resin was washed 5 times with NMP.

GP3 Standard Amino Acid Coupling

A solution of Fmoc-Xaa-OH (2 eq.), 2-(1*H*-Benzotriazole-1-yl)-1,1,3,3-tetramethyluronium tetrafluoroborate (TBTU) (2 eq.), Hydroxybenzotriazole (HOBt; 2 eq.), and DIPEA (5.2 eq.) in NMP (1 mL/g resin) was added to the resin-bound free amine peptide and shaken for 60 min at room temperature and washed 5 times with NMP.

GP4 On-Resin *N-Methylation*

The linear, Fmoc-deprotected peptide is washed with DCM (3x) incubated with a solution of 2-nitrobenzenesulfonylchloride (*o*-NBS-Cl, 4 eq.) and 2,4,6-collidine (10 eq.) in DCM for 20 min at r.t.. The resin is washed with DCM (3x) and THF *abs*. (5x). A solution containing PPh_3 (5 eq.) and MeOH *abs.* (10 eq.) in THF *abs.* is added to the resin. DIAD (5 eq.) in a small amount THF *abs*. is added stepwise to the resin and the solution is incubated for 15 min and washed with THF (5x) and NMP (5x). For *o*-Ns deprotection, the resin-bound *o*-Ns-peptides were stirred in a solution of mercaptoethanol (10 eq.) and DBU (5 eq.) in NMP (1 mL/g resin) for 5 min. The deprotection procedure was repeated one more time and the resin was washed 5 times with NMP.

GP5 Cleavage of linear peptides from the resin

For complete cleavage from the resin the peptides were treated three times with a solution of DCM and hexafluoroisopropanol (HFIP; 4:1; v:v) at room temperature for half an hour and the solvent evaporated under reduced pressure.

GP6 Backbone cyclization with DPPA

To a solution of peptide in DMF (1 mM peptide concentration) and $NaHCO_3$ (5 eq.) diphenylphosphoryl azide (DPPA; 3 eq.) was added at room temperature (RT) and stirred overnight or until no linear peptide could be observed by ESI-MS. The solvent was evaporated to a small volume under reduced pressure and the peptides precipitated in saturated NaCl solution and washed two times in HPLC grade water.

GP7 Removal of Acid Labile Side Chain Protecting Groups

Cyclized peptides were stirred in a solution of TFA, water and TIPS (triisopropyl silane) (95:2.5:2.5) at room temperature for one hour or until no more protected peptide could be observed by ESI-MS and precipitated in diethylether and washed two more times.

GP8 Cyclization via disulfide-bridges

The HPLC-purified peptide is dissolved in a mixture of DMSO and H_2O (1/1, v/v) and vigorously stirred until no starting material could be detected in LC-MS. The solvent was removed under reduced pressure (co-evaporation with $CHCl_3$), taken up in a mixture of ACN/H_2O and lyophilized.

Ligand synthesis analytical data

.

	synthesis	supplier/ donor	order number	t_R (min)**	mass (m/z)
Echistatin	no	Sigma-Aldrich	E1518	-	-
RGD	no	APexBio	B3708	-	-
RGDS	no	GenScript	RP10861	-	-
GRGDS	no	AnaSpec	AS-61107	-	-
GRGDSP	no	AnaSpec	AS-22945	-	-
GRGDSPK	no	AnaSpec	61109	-	-
GRGDNP	no	AnaSpec	62049	-	-
GRGDTP	no	AnaSpec	22947	-	-
c(RGDfV)	yes	-	-	5.8	575.4
Cilengitide c(RGDf(NMe)V)	yes	-	-	6.6	589.5
c(RGDfK)	yes	-	-	5.1	603.4
c(RGDyK)	no	AnaSpec	61183-1	-	
c(RGDfC)	no	AnaSpec	63785-1	-	
sn243	yes	-	-		522.5
RGD-4C	no	Abbiotech	350363		
RGD10 (RGD-2C)	yes	-	-	4.3	1476.7
NC100717	no	GE	donation		
c(phgisoDGRk)	yes	-	-	4.4	590.4
44b	yes	-	-	6.7	513.5
ATN161	no	MedKoo	200350a	-	-
JSM6427	no	3B Pharma-ceuticals	-	-	-
RTDLDSLRT	yes	-	-	4.9	1077.8
A20FMDV2	no	Dr. Curnis / Prof. Corti	donation		
Mol11*	yes	-	-		525.5
c(FRGDLAFp(NMe)K(Ac)	yes	-	-	6.3	1088.5
Eptifibatide	no	Tocris	4725		

Tirofiban	no	Sigma	30165	
GR144053	no	TOCRIS	1263	-

* (*S*-Enantiomer)

** HPLC-MS (ESI), C18-column, gradient 10-90% ACN/H_2O

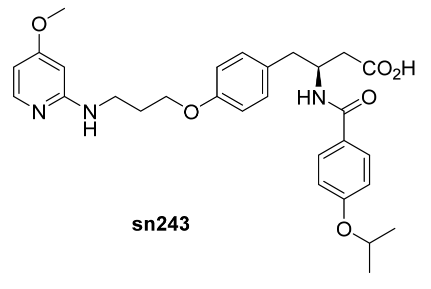

sn243

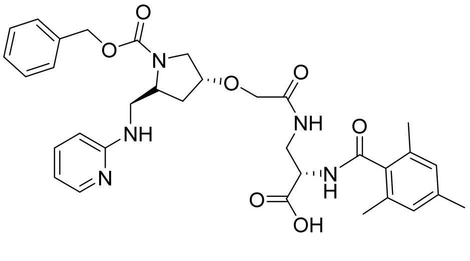

JSM-6427

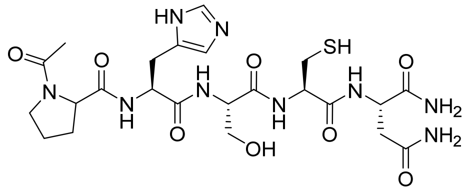

ATN-161

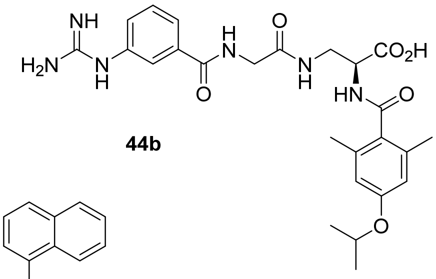

44b

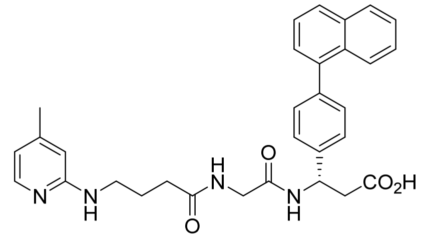

Mol11

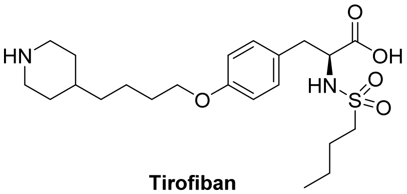

Tirofiban

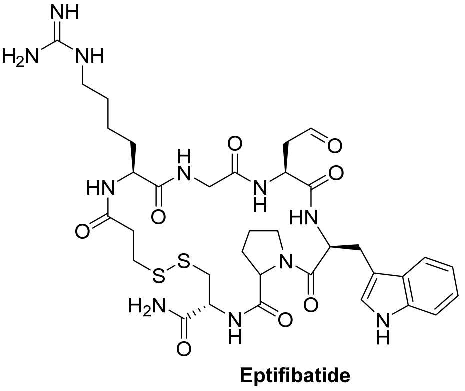

Eptifibatide

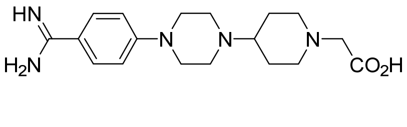

GR144053

Synthesis of *sn243* and *44b*

The synthesis was done as described in Rechenmacher et al. *Angew. Chem. Int. Ed.* **2013**, *52*, 1572-1575.

Synthesis of Mol11

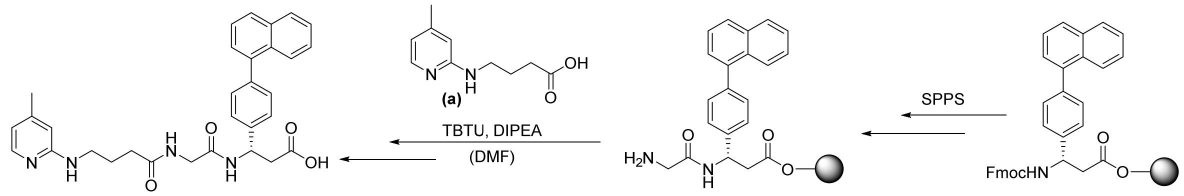

The enantiomerically pure compound (*S*)-3-(Fmoc-amino)-3-(4-(naphthalen-1-yl)phenyl) propionic acid (IRIS Biotech) was loaded to the CTC resin analog to **GP1**. After Fmoc-deprotection (**GP2**), Fmoc-Gly-OH was coupled on resin (**GP3**). In the next step, the building block **(a)** was coupled followed by a cleavage from the resin. After evaporation of the solvent, Mol11 was purified by semipreparative HPLC.

Synthesis of (a)

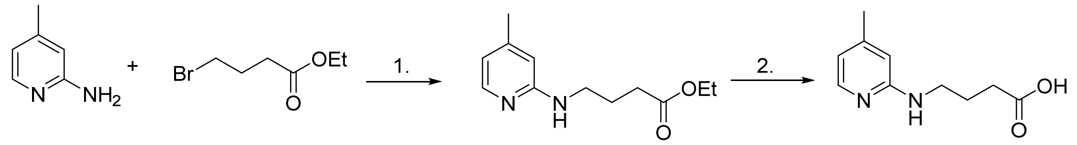

2-amino-4-picolin (11.9 g, 110 mmol, 1.0 eq) is mixed with Na_2CO_3 (11.7 g, 110 mmol, 1.0 eq) and 4-bromo ethyl butyrat is slowly added under stirring. The mixture is stirred for 5 h at 120°C and subsequently taken up in 200 ml H_2O. Afterwards, 1M HCl is used to adjust the pH = 8. After extraction with 100 ml EtOAc (2x), the organic layer is dried using Na_2SO_4 and the solvent removed under vacuo. Flash chromatography ($CHCl_3$/MeOH: 95/5 (v/v), R_f = 0.4) yields methyl-4-((4-methylpyridin-2-yl))butanoate (3.6 g) as a brown solid.
This compound (3.6 g) was dissolved in 50 ml dioxan and 20 ml of a 2M aqueous NaOH-solution was added and stirred over night. Dioxan is removed under vacuo, 100 ml H_2O added and the extraction with EtOAc done. The title compound is dissolved in the water phase, which is lyophilized over night. Flash chromatography ($CHCl_3$/MeOH/AcOH: 85/10 /5 (v/v/v), R_f = 0.28) yields **(a)** (2.8 g 14.4 mmol, 13%) brownish solid.

^{1}H-NMR: (300 MHz, DMSO-*d*$_6$, 300 K) δ = 7.75 (d, *J* = 5.5 Hz, 1H), 6.44 (s, 1H), 6.35 (dd, *J* = 5.6 Hz, *J* = 1.4 Hz, 1H), 3.21 (dt, *J* = 6.4 Hz, *J* = 6.6 Hz, 2H), 2.80 (t, *J* = 7.5 Hz, 2H), 2.13 (s, 3H), 1.70 (quin, *J* = 7.2 Hz, 2H). **MS (ESI):** m/z: 194.1 $[m+H]^+$. **HPLC** (10-90 % 7 min) t_R = 1.22 min.

Functionalized compounds

	synthesis	supplier /donor	order number	t_R (min)	mass (m/z)
**F-Galacto-*c*(RGDfK)**	no	ABX	9800		
**(Ga)NOPO-*c*(RGDfK)**	no	Dr. Notni / Prof. Wester	donation	-	-
***c*(RGDfK)-Hegas-MPA**	yes			8.7	1027.7
***c*(phg-*iso*DGR-k)-O2Oc-MPA**	yes	-	-	4.2	823.5
**Flucilatide**	no	GE	donation	-	-

** HPLC-MS (ESI), C18-column, gradient 10-90% ACN/H_2O, 15 min

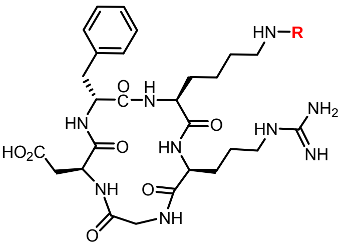

c(RGDfK)

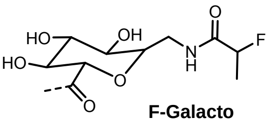

F-Galacto

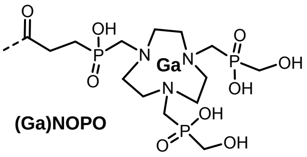

(Ga)NOPO

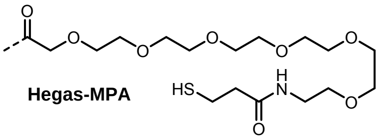

Hegas-MPA

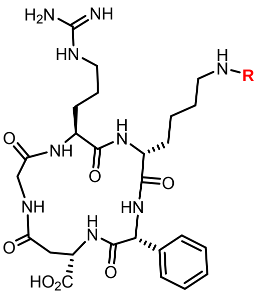

c(phgisoDGRk)

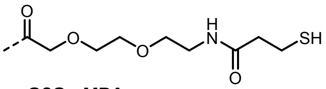

O2Oc-MPA

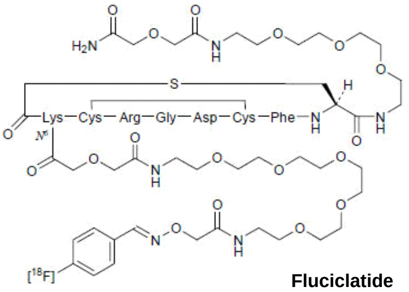

Fluciclatide

Synthesis of *c*(RGDfK)-Hegas-MPA

The orthogonally protected cyclic peptide *cyclo*(Arg(Pbf)-Gly-Asp(*t*Bu)-D-Phe-Lys(Cbz)) was synthesized following the general procedures outlined above. This compound was orthogonally Cbz-deprotected by dissolving the compound in MeOH, adding catalytic amounts (20 mol%) of Pd/C and stirring over night in an H_2-atmosphere (1 atm.). The catalyst is filtered off and the solvent removed under reduced pressure and the remaining compound (***c(R(Pbf)GD(tBu)fK***)) taken up in a mixture of ACN/H_2O and lyophilized over night.

In parallel, Fmoc-21-amino-4,7,10,13,16,19-hexaoxaheneicosanoic acid is loaded to the resin according to **GP1** and subsequently coupled after Fmoc-deprotection (**GP2**) on resin to 3-mercapto (trityl) propionic acid (**GP3**). After cleavage from the resin (**GP5**), the compound ***Hegas-MPA*** is lyophilized from a ACN/H_2O-mixture).

1.2 eq. of ***Hegas-MPA***, 1.2 eq. HATU and 3.0 eq. DIPEA are dissolved in a minimal amount of DMF (app. 30 mM) and stirred for 10 min at RT. Afterwards, a solution of ***c(R(Pbf)GD(tBu)fK*** is added dropwise to this solution and stirred for 1 h at RT. After removing the solvent in vacuo, the peptide conjugate gets directly deprotected by treating the crude product with a mixture of TFA/DCM/H_2O (80/15/5, v/v/v) for 20 min. The title compound is obtained after semipreparative HPLC-purification.

Synthesis of *c*(phg-*iso*DGR-k)-O2Oc-MPA

The orthogonally protected cyclic peptide *cyclo*(D-Phg-*iso*Asp(*t*Bu)-Gly-Arg(Pbf)-D-Lys(Cbz)) was synthesized following the general procedures outlined above. This compound was orthogonally Cbz-deprotected by dissolving the compound in MeOH, adding catalytic amounts (20 mol%) of Pd/C and stirring over night in an H_2-atmosphere (1 atm.). The catalyst is filtered off and the solvent removed under reduced pressure and the remaining compound (***c(phg-isoDGR(Pbf)k***)) taken up in a mixture of ACN/H_2O and lyophilized over night.

In parallel, 8-Fmoc-3,6-dioxaoctanoic acid is loaded to the resin according to **GP1** and subsequently coupled after Fmoc-deprotection (**GP2**) on resin to 3-mercapto (trityl) propionic acid (**GP3**). After cleavage from the resin (**GP5**), the compound ***O2Oc-MPA*** is lyophilized from a ACN/H_2O-mixture).

1.2 eq. of ***O2Oc-MPA***, 1.2 eq. HATU and 3.0 eq. DIPEA are dissolved in a minimal amount of DMF (app. 30 mM) and stirred for 10 min at RT. Afterwards, a solution of ***c(phg-isoDGR(Pbf)k)*** is added dropwise to this solution and stirred for 1 h at RT. After removing the solvent *in vacuo*, the peptide conjugate gets directly deprotected by treating the crude product with a mixture of TFA/DCM/H_2O (80/15/5, v/v/v) for 20 min. The title compound is obtained after semipreparative HPLC-purification.

Integrin Binding Assay

The activity and selectivity of integrin ligands were determined by a solid-phase binding assay according to the previously reported protocol [i] using coated extracellular matrix proteins and soluble integrins. The following compounds were used as internal standards: Cilengitide, *c*(-RGDf(*N*Me)V-) (αvβ3 – 0.54 nM, αvβ5 – 8 nM, α5β1 – 15.4 nM), linear peptide RTDLDSLRT[4] (αvβ6 – 33 nM; αvβ8 – 100 nM) and tirofiban[5] (αIIbβ3 – 1.2 nM).

Flat-bottom 96-well ELISA plates (BRAND, Wertheim, Germany) were coated overnight at 4 °C with the **ECM-protein (1)** (100 µL per well) in carbonate buffer (15 mM Na_2CO_3, 35 mM $NaHCO_3$, pH 9.6). Each well was then washed with PBS-T-buffer (phosphate-buffered saline/Tween20, 137 mM NaCl, 2.7 mM KCl, 10 mM Na_2HPO_4, 2 mM KH_2PO_4, 0.01% Tween20, pH 7.4; 3 × 200 µL) and blocked for 1 h at room temperature with TS-B-buffer (Tris-saline/BSA buffer; 150 µL/well; 20 mM Tris-HCl, 150 mM NaCl, 1 mM $CaCl_2$, 1 mM $MgCl_2$, 1 mM $MnCl_2$, pH 7.5, 1% BSA). In the meantime, a dilution series of the compound and internal standard is prepared in an extra plate, starting from 20 µM to 6.4 nM in 1:5 dilution steps. After washing the assay plate three times with PBS-T (200 µL), 50 µL of the dilution series were transfered to each well from B – G. Well A was filled with 100 µL TSB-solution (blank) and well H was filled with 50 µL TS-B-buffer. 50 µL of a solution of **human integrin (2)** in TS-B-buffer was transfered to wells H – B and incubated for 1 h at rt. The plate was washed three times with PBS-T buffer, and then **primary antibody (3)** (100 µL per well) was added to the plate. After incubation for 1 h at rt, the plate was washed three times with PBS-T. Then, **secondary peroxidase-labeled antibody (4)** (100 µL/well) was added to the plate and incubated for 1 h at rt. After washing the plate three times with PBS-T, the plate was developed by quick addition of SeramunBlau (50 µL per well, Seramun Diagnostic GmbH, Heidesee, Germany) and incubated for 5 min at rt in the dark. The reaction was stopped with 3 M H_2SO_4 (50 µL/well), and the absorbance was measured at 450 nm with a plate reader (POLARstar Galaxy, BMG Labtechnologies). The IC50 of each compound was tested in duplicate, and the resulting inhibition curves were analyzed using OriginPro 7.5G software. The inflection point describes the IC50 value. All determined IC50 were referenced to the activity of the internal standard.

<u>αvβ3</u>

(1) 1.0 µg/mL human vitronectin; Millipore
(2) 2.0 µg/mL, human αvβ3-integrin, R&D
(3) 2.0 µg/mL, mouse anti-human CD51/61, BD Biosciences
(4) 1.0 µg/mL, anti-mouse IgG-POD, Sigma-Aldrich

α5β1

(1) 0.5 μg/mL; human fibronectin, Sigma-Aldrich
(2) 2.0 μg/mL, human α5β1-integrin, R&D
(3) 1.0 μg/mL, mouse anti-human CD49e, BD Biosciences
(4) 2.0 μg/mL, anti-mouse IgG-POD, Sigma-Aldrich

αvβ5

(1) 5.0 μg/mL; human vitronectin, Millipore
(2) 3.0 μg/mL, human αvβ5-integrin, Millipore
(3) 1:500 dilution, anti-αv mouse anti-human MAB1978, Millipore
(4) 1.0 μg/mL, anti-mouse IgG-POD, Sigma-Aldrich

αvβ6

(1) 0.4 μg/mL; LAP (TGF-β), R&D
(2) 0.5 μg/mL, human αvβ6-Integrin, R&D
(3) 1:500 dilution, anti-αv mouse anti-human MAB1978, Millipore
(4) 2.0 μg/mL, anti-mouse IgG-POD, Sigma-Aldrich

αvβ8

(1) 0.4 μg/mL; LAP (TGF-b), R&D
(2) 0.5 μg/mL, human αvβ8-Integrin, R&D
(3) 1:500 dilution, anti-αv mouse antihuman MAB1978, Millipore
(4) 2.0 μg/mL, anti-mouse IgG-POD, Sigma-Aldrich

αIIbβ3

(1) 10.0 μg/mL; human fibrinogen, Sigma-Aldrich
(2) 5.0 μg/mL, human platelet integrin αIIbβ3, VWR
(3) 2.0 μg/mL, mouse anti-human CD41b, BD Biosciences
(4) 1.0 μg/mL, anti-mouse IgG-POD, Sigma-Aldrich

---

[i] A.O. Frank, E. Otto, C.Mas-Moruno, H.B. Schiller, L. Marinelli, S. Cosconati, A. Bochen, D. Vossmeyer,, G. Zahn, R. Stragies, E. Novellino, H. Kessler. *Angew. Chem. Int. Ed.* **2010**, *49*, 9278−9281.
